# Serum CXCL13 reflects local B-cell mediated inflammatory demyelinating peripheral neuropathy

**DOI:** 10.1038/s41598-019-52643-2

**Published:** 2019-11-11

**Authors:** Young Hee Kim, So Young Jang, Yoon Kyung Shin, Young Rae Jo, Byeol-A. Yoon, Soo Hyun Nam, Byung-Ok Choi, Ha Young Shin, Seung Woo Kim, Se Hoon Kim, Jong Kuk Kim, Hwan Tae Park

**Affiliations:** 1 Peripheral Neuropathy Research Center (PNRC), Dong-A University College of Medicine, Busan, 49201 Republic of Korea; 2 Department of Molecular Neuroscience, Dong-A University College of Medicine, Busan, 49201 Republic of Korea; 3 Department of Neurology, Dong-A University College of Medicine, Busan, 49201 Republic of Korea; 4 Department of Neurology, Sungkyunkwan University School of Medicine, Seoul, 06351 Republic of Korea; 5 Stem Cell & Regenerative Medicine Institute, Samsung Medical Center, 81 Irwon-ro, Gangnam-gu, Seoul 06351 Republic of Korea; 6 Department of Neurology, Yonsei University College of Medicine, 50-1 Yonsei-ro, Seodaemun-gu, Seoul 03772 Republic of Korea; 7 Department of Pathology, Yonsei University College of Medicine, 50-1 Yonsei-ro, Seodaemun-gu, Seoul 03772 Republic of Korea

**Keywords:** Neuroimmunology, Acute inflammation

## Abstract

Immune damages on the peripheral myelin sheath under pro-inflammatory milieu result in primary demyelination in inflammatory demyelinating neuropathy. Inflammatory cytokines implicating in the pathogenesis of inflammatory demyelinating neuropathy have been used for the development of potential biomarkers for the diagnosis of the diseases. In this study, we have found that macrophages, which induce demyelination, expressed a B-cell-recruiting factor CXC chemokine ligand 13 (CXCL13) in mouse and human inflammatory demyelinating nerves. The serum levels of CXCL13 were also higher in inflammatory demyelinating neuropathic patients but not in acute motor axonal neuropathy or a hereditary demyelinating neuropathy, Charcot-Marie-Tooth disease type 1a. In addition, CXCL13-expressing macrophages were not observed in the sciatic nerves after axonal injury, which causes the activation of innate immunity and Wallerian demyelination. Our findings indicate that the detection of serum CXCL13 will be useful to specifically recognize inflammatory demyelinating neuropathies in human.

## Introduction

Inflammatory peripheral neuropathy encompasses various types of peripheral neuropathies associated with nerve inflammation, including Guillain-Barré syndrome (GBS) and chronic inflammatory demyelinating polyradiculoneuropathy (CIDP)^1^. GBS is a representative type of acute autoimmune peripheral neuropathy that results in respiratory weakness and has a fatal course in certain cases^2^. GBS includes acute inflammatory demyelinating polyradiculoneuropathy (AIDP), acute motor axonal neuropathy (AMAN), and Miller Fisher syndrome based on clinical and electrophysiological findings. The pathomechanism of AMAN is well known to be related with a specific preceding infection and several type of anti-ganglioside antibodies^3^. The discovery of anti-ganglioside antibodies has provided not only diagnostic, but also mechanistic insights into the development of AMAN^4^. In contrast, even though auto-antibodies against nodal/paranodal proteins such as neurofascin or contactin have been found in some cases of CIDP, it is still unclear how most AIDP and CIDP can occur, and no clinically applicable serum biomarkers of AIDP and CIDP have been developed to date^1,5^.

Inflammatory peripheral demyelination is caused by immunological attacks on the myelin sheath or Schwann cell (SC), the myelin-forming glial cell. In inflammatory demyelination, multiple secretory molecules, including inflammatory cytokines, are expressed by immune cells and dedifferentiated SCs^6–11^, and these proteins may be potential targets for biomarker development. SC dedifferentiation indicates the phenotype transition of a mature SC into an immature SC and is found during Wallerian degeneration (WD) after axonal injury^12,13^. During WD, dedifferentiated SC evokes innate immunity by secreting several cytokines including TNF-a^14,15^. Thus, the identification of specific cytokines expressed only in inflammatory demyelination, but not in Wallerian demyelination, may be helpful to develop biomarkers delineating inflammatory demyelination.

In the present study, we compared cytokine expression profiles between inflammatory versus Wallerian demyelination, and found that a local activation of the CXC chemokine ligand 13 (CXCL13)-CXC chemokine receptor 5 (CXCR5) immune pathway only in inflammatory demyelinating nerves. The inflammatory demyelination-specific expression of CXCL13 in the mouse and human peripheral nerves was represented in the sera of inflammatory demyelinating neuropathy patients, but not in the sera of AMAN and CMT1a patients, indicating the potential diagnostic utility of CXCL13 in inflammatory demyelinating peripheral neuropathy.

## Results

### Pro-inflammatory milieu in inflammatory demyelinating nerves contains CXCL13+ macrophages

**Figure 1:**
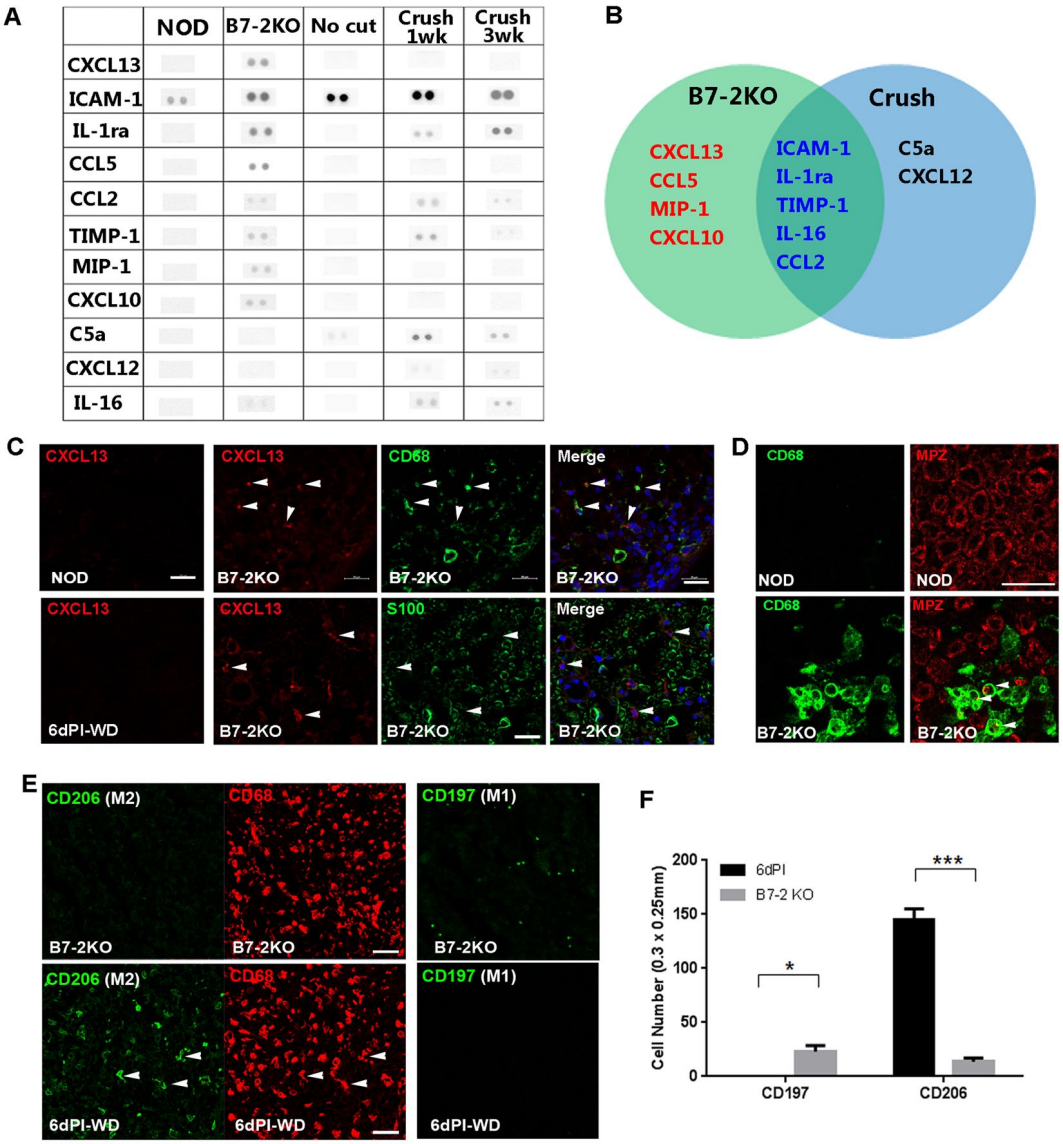
M1-macrophage-related pro-inflammatory environment with CXC chemokine ligand 13 expression in B7-2KO nerves. (**A**) Representative blots of cytokine arrays in B7-2KO nerves and injured nerves with their respective controls. (B) Diagram of the cytokine expression profiles in B7-2KO nerves and injured C57BL/6 nerves. The CXC chemokine ligand 13 (CXCL13), C-C chemokine ligand 5 (CCL5), macrophage inflammatory protein 1 (MIP-1), and CXCL10 levels were specifically increased in B7-2KO nerves. (**C**) CXCL13 expression was induced in CD68^+^ macrophages (arrowheads), but not in S100-positive SCs, in B7-2KO nerves. There were no CXCL13-positive cells in NOD nerves or injured C57BL/6 nerves (6dPI, 6 days post-injury). WD, Wallerian degeneration. Scale bar, 20 μm. (**D**) IF staining showing the association of macrophage infiltration (CD68) and demyelination (MPZ; myelin protein zero). Arrowheads; demyelinating macrophages. Scale bar, 20 μm. (**E**) Antibodies against CD206 and CD197 were employed to detect M2 and M1 macrophages, respectively, in nerve sections. In B7-2KO nerves, most CD68^+^ macrophages were CD206^−^, while some CD197^+^ cells were present. During WD, most CD68^+^ macrophages were CD206^+^ (arrowheads), while only a few cells were CD197^+^. Scale bar, 20 μm. (F) Quantification of CD206^+^ and CD197^+^ cells in a unit area (300 μm × 250 μm) of sciatic nerve sections. Unpaired Student’s *t*-test; ***p < 0.001.

To identify pro-inflammatory condition-specific cytokines expressed in demyelinating SCs, we compared cytokine expression profiles in the neuropathic sciatic nerves of B7-2 knockout NOD (B7-2KO) mice, that show a spontaneous autoimmune demyelinating neuropathy^8,16^ and injured C57BL/6 nerves (in this case, the innate immunity is activated during WD) with a commercial cytokine expression panel^17^. The expression levels of intercellular adhesion molecule-1 (ICAM-1), interleukin-1 receptor antagonist, tissue inhibitor of metalloproteinases 1, C-C chemokine ligand 2 (CCL2), and IL-16 were increased in the sciatic nerves of both B7-2KO mice and injured C57BL/6 mice compared to each control of both conditions (Fig. 1A,B). We found that CCL5, macrophage inflammatory protein-1, and CXCL10 were specifically upregulated in B7-2KO nerves compared to the NOD control nerves and also to the injured C57BL6 nerves (Fig. 1A,B). In consistency with this result, CCL5 and CXCL10 are INF-γ inducible type-1 macrophage (M1)-related cytokines and both of them are also known to be involved in inflammatory neuropathy^11,18^. Interestingly, CXCL13, a B-cell-recruiting factor, which is constitutively expressed in follicular cells and macrophages in secondary lymphoid tissues^19^, was up-regulated selectively in B7-2KO nerves, but not in the injured sciatic nerves (Fig. 1A,B).

In accordance with the cytokine array data, IF staining revealed numerous CXCL13-positive cells in the B7-2KO nerves, but not in NOD nerves (Fig. 1C). Double immunostainings for CXCL13 and the macrophage marker CD68 or the SC marker S100 revealed that CXCL13 expression was limited to the CD68^+^ macrophages in the B7-2KO nerves. In contrast, even though numerous infiltrating CD68^+^ macrophages were present in the injured C57BL/6 nerves, no CXCL13-positive cells were observed, suggesting that the pro-inflammatory condition in the B7-2KO nerves might be related to the expression of CXCL13 in macrophages (Fig. 1C,E). In addition, we examined the relation of myelin damage to the macrophage localization in B7-2KO nerves using immunostaining, and found a substantial regional correlation of the infiltration of macrophages and demyelination in B7-KO nerves (Fig. 1D). Thus, macrophage infiltration with CXCL13 expression appeared to be associated with demyelination.

To evaluate the polarization of macrophages into M1 and/or M2 is related to the expression of CXCL13 in B7-2KO nerves, IF staining with antibodies against M1 and M2 macrophage markers was performed on sciatic nerve sections (Fig. 1E). In B7-2KO nerves, while few CD68^+^ macrophages expressed CD206, an M2 marker, numerous macrophages positive for the M1 marker, CD197, were present. In contrast, the sciatic nerves undergoing WD exhibited high numbers of CD206^+^ macrophages without CD197 expression (Fig. 1E,F), suggesting that inflammatory polarization of macrophages in B7-2KO nerves may be associated with CXCL13 expression. Taken together, these results indicate that demyelinating B7-2KO nerves exhibit pro-inflammatory milieu, which is related to the selective CXCL13 expression and inflammatory demyelination.

### Local CXCL13/CXCR5-mediated immune reaction in inflammatory demyelinating nerves

**Figure 2:**
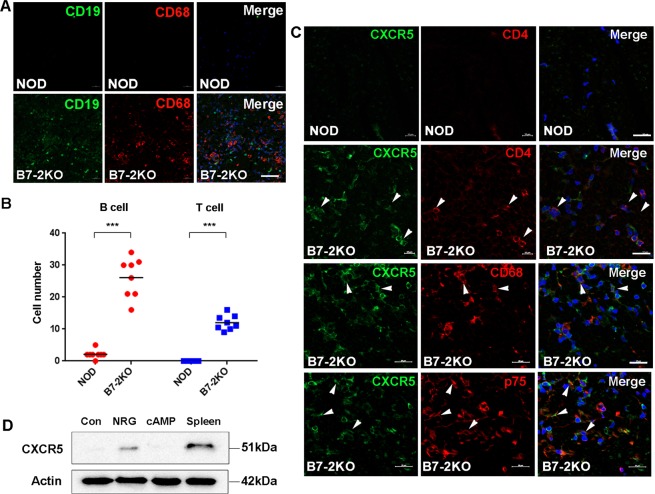
The expression of CXC chemokine receptor 5 in B7-2KO nerves. (**A**) IF staining showing the infiltration of B cells, which were stained with an antibody against CD19 (green), in association of macrophage infiltration (CD68) in B7-2KO nerves. Scale bar, 50 μm. (**B**) The numbers of B- and T-cells in B7-2KO sciatic nerve and non-obese diabetic (NOD) control nerve. A dot indicates the number in a sciatic nerve section. Unpaired Student’s *t*-test; ***p < 0.001. (**C**) The profile of CXCR5 expression in B7-2KO nerves. Double immunostaining for CXCR5 and specific markers of T-cells (CD4), macrophages (CD68), and dedifferentiated Schwann cells (p75) showed that all of these cell types had increased CXCR5 levels in B7-2KO nerves. Arrowheads indicate double-positive cells. Scale bar, 20 μm. (**D**) Western blot analysis showed that cultured primary SCs expressed CXCL5 upon exposure to neuregulin (NRG). Spleen was used as a positive control. The CXCL5 image was cropped from a full length gel (Supplementary Fig. 1).

The expression of CXCL 13 in B7-2KO nerves may indicate local B-cell-related immune activation via CXCR5, the receptor of CXCL13. We thus examined the infiltration of CD19^+^ B-cells in B7-2KO and NOD nerves, and found a high number of infiltrating B-cells in the former but not the latter (Fig. 2A,B). We next examined the cellular localization of CXCR5 in B7-2KO nerves using IF staining. Low levels of CXCR5 expression were found in the axons and SCs of NOD nerves, whereas CXCR5 expression was dramatically increased in B7-2KO nerves. Double immunostaining revealed that many of the CXCR5-positive mononuclear cells in the B7-2KO nerves were CD4^+^ T cell and CD68^+^ macrophages (Fig. 2B,C). In addition, some p75-positive dedifferentiated SCs also expressed CXCR5 in the B7-2KO nerves (Fig. 2C). The expression of CXCR5 in SCs was confirmed by western blot analysis in cultured primary SCs and the expression was further increased by the treatment of neuregulin, a SC dedifferentiating factor, but not by dibutyryl-cAMP, a SC differentiating reagent (Fig. 2D). These findings suggest that CXCL13 and CXCR5^+^/CD4^+^ T cell, B-cell and SC may be pathologically implicated in the mouse autoimmune inflammatory demyelination.

### Increased serum CXCL13 levels may specifically reflect inflammatory peripheral demyelination

**Figure 3:**
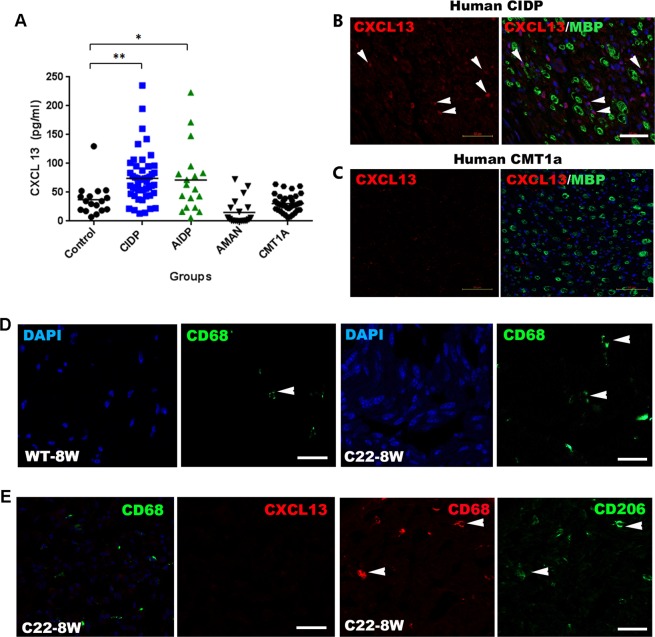
Serum CXC chemokine ligand 13 (CXCL13) levels are increased in inflammatory demyelinating neuropathy. Serum CXCL13 concentrations in peripheral neuropathy patients were examined using ELISA. The serum levels of CXCL13 were significantly increased in patients with acute (AIDP; *p* < 0.05) and chronic (CIDP; *p* < 0.01) inflammatory demyelinating polyradiculoneuropathy compared to those in healthy controls, acute motor axonal neuropathy (AMAN), and Charcot-Marie-Tooth type 1a (CMT1a) patient groups. Significant differences between patient groups and healthy controls by repeated-measures ANOVA are denoted by *(p < 0.05) and **(p < 0.01). (**B**,**C**) CXCL13 expression in the sural nerves of human CIDP (**B**) and CMT1a patients (**C**). Arrows indicate CXCL13^+^ cells in the sural nerves. The sural nerve of a CMT1a patient exhibited hypomyelinated nerves without noticeable CXCL13 expression. Scale bar, 50 μm. (**D**) Minimal CD68^+^ macrophages infiltration (arrowheads) in C22 nerves. Scale bar, 20 μm. (**E**) C22 sciatic nerves exhibited minor infiltration of macrophages (CD68, arrowheads), however they did not show CXCL13 expression. Most of CD68^+^ macrophages were immunoreactive for CD206 (arrows) in C22 nerves.

We next examined serum CXCL13 levels in peripheral neuropathy patients. We found higher levels of CXCL13 in the sera of CIDP (71.41 ± 5.83 pg/mL, p < 0.01) and AIDP (71.34 ± 13.73 pg/mL, p < 0.05) patients compared with those in the control sera (37.35 ± 6.75 pg/mL). In contrast, the CXCL13 levels in the sera of AMAN (17.99 ± 7.02 pg/mL) and CMT1a (47.39 ± 3.98 pg/mL) patients were not significantly different from the control levels (p > 0.05; Fig. 3A). In consistency with ELISA data, we found CXCL13+ cell infiltration in the sural nerves of CIDP patients, but not in a CMT1a patient (Fig. 3B,C).

Recently, it was demonstrated that macrophage infiltration is related to the pathogenesis of hereditary demyelinating neuropathy^20^. We thus examined a potential expression of CXCL13^+^ macrophages in the sciatic nerves of C22 mice, an animal model of CMT1a, and found that most macrophages were CD206-positive but CXCL13-negative (Fig. 3D,E), which was in line with the normal serum levels of CXCL13 in the CMT1a patients (Fig. 3A). Taken together, our findings indicate that CXCL13^+^ macrophages may contribute to the high serum of levels of CXCL13 in inflammatory demyelinating neuropathy patients.

## Discussion

Autoantibodies against the myelin sheath and nodal/paranodal proteins have been considered to play a role in the pathologic demyelination in autoimmune peripheral neuropathies^1,5^. An anti-myelin protein zero antibody was also found in B7-2KO mice^21^. Even though these tissue-specific auto-antibodies are considered to be generated by B-cell activation in the secondary lymphoid organs, recent studies concerning several autoimmune diseases suggest that a local immune reaction mediated by B- and T-cells, as well as antigen-presenting cells (APCs), in the primary inflammatory lesion plays a role in the disease pathogenesis^22^. In particular, cytokines related to the survival, proliferation, and tolerance of B-cells may induce abnormal B-cell responses in the lesion sites. For example, the expression of CXCL13, which is essential for the development of secondary lymphoid organs^19^, and B-cell-activating factor within the central nervous system are implicated in the development of experimental autoimmune encephalitis^22,23^. In the present study, we showed for the first time that CXCL13 is specifically induced by macrophages in the mouse and human inflammatory demyelinating peripheral nerves but not in nerves undergoing WD and of mouse and human CMT1a. We also determined the localization of CXCR5, the receptor of CXCL13, in immune cells and SCs of B7-2KO nerves. The induction of CXCR5 in SCs of B7-2KO nerves indicates that pro-inflammatory conditions involving CXCL13 activation may regulate SC physiology through CXCR5. Considering the potential role of SCs as APCs^24^, these findings suggest that the CXCL13/CXCR5-mediated local immune reaction involving SCs may contribute to the development of autoimmune reaction in inflammatory demyelinating neuropathy.

Based on the high correlation of increased CXCL13 serum levels with the development of autoimmune diseases, CXCL13 has been considered as a potential biomarker of various autoimmune diseases such as Sjogren’s syndrome^25^. We found elevated serum levels of CXCL13 in AIDP and CIDP patients compared with those in healthy controls and CMT1a patients. CXCL13 appears to be more specific for representing inflammatory demyelinating neuropathy compared with the previously demonstrated cytokines such as HGF, TNF-α and CCLs for several reasons. Firstly, as revealed in B7-2KO mice, CXCL13 may be involved in the pro-inflammatory condition associated with local immune activation within neuropathic nerves. Secondly, even though HGF, TNF-α and CCLs may represent the local inflammatory condition in nerves, these cytokines could be induced as a result of the activation of innate immunity during WD^6,26,27^; hence, they cannot specifically represent the pro-inflammatory condition in nerves. Finally, we found no significant increase in CXCL13 serum levels in patients with AMAN, an axonal form of GBS. The antigen-mimetic pathophysiology of AMAN is relatively well established: *Campylobacter jejuni* infection results in B-cell activation in the secondary lymphoid organs, leading to the generation of antibodies against glycolipids of the infective microorganism attacking peripheral nerve axons^2,3^. The absence of CXCL13 induction in AMAN indicates that local immune activation through the CXCL13-CXCL5 pathway may not be involved in the development of AMAN, consistent with the previously proposed theory of antibody-complement-mediated axonal injury being responsible for AMAN, which is distinct from AIDP and CIDP.

In conclusion, our findings suggest that the concurrent induction of CXCL13 serum levels could be a pathology-relevant and disease-specific biomarker of inflammatory demyelinating peripheral neuropathy.

## Materials and Methods

All methods used in this study followed the guidelines and regulations by the Dong-A University Research Ethics Committee.

### Antibodies and reagents

Antibodies against β-actin, p75 neurotrophin receptor (p75), CD68, CD4, and myelin basic protein (MBP) were purchased from Santa Cruz Biotechnology (Santa Cruz, CA, USA). The antibody against human CXCL13 was purchased from R&D Systems (Minneapolis, MN, USA). Antibodies against CD206 and CXCR5 were obtained from Abcam (Cambridge, UK), and Alexa-Fluor 488 conjugated CD197 antibody was purchased from Biolegend (San Diego, CA, USA). Antibodies against CXCL13 and myelin basic protein were obtained from Thermo Fisher Scientific (Waltham, MA, USA). Horseradish peroxidase (HRP)-linked anti-rabbit IgG and anti-mouse IgG were obtained from Cell Signaling technology (Danvers, MA, USA). Alexa Fluor 488 or Cy3-conjugated secondary antibodies were purchased from Molecular probes (Carlsbad, CA, USA). Every recombinant cytokine used in this study was obtained from Peprotech (Rocky Hill, NJ, USA) and R&D Systems. Unless otherwise specified, all other reagents were purchased from Sigma–Aldrich (St. Louis, MO, USA).

### Animals

All the methods for animal surgery followed the guidelines and regulations approved by the Dong-A University Committee on animal research which follows the guidelines for animal experiments that were established by the Korean Academy of Medical Sciences (No. DIACUC-16-21). Non-obese diabetic (NOD) and NOD-B7-2 knockout (B7-2KO) mice were purchased from Jackson Lab (Stock No. 004762, Bar Harbor, ME, USA). The genotypes were determined and neuropathy was assessed weekly from 20 weeks after birth by examining tail-drop and hind-limb paralysis as we previously described^28^. The clinical progression of motor deficits was divided into 5 grades: grade (G) 0, no signs; G1, floppy tail; G2, mild paraparesis or unilateral hindlimb paralysis; G3, severe paraparesis; G4, tetraparesis; G5, moribund condition or death. PMP22 transgenic mice (C22)^29^ were obtained from Samsung Medical Center (Seoul, Korea). The mouse model contains seven copies of human *Peripheral myelin protein 22* (PMP22) gene leading to a demyelinating neuropathy.

For a sciatic nerve injury, left sciatic nerves of adult C57BL/6 mice were axotomized 5 mm proximal to the tibioperoneal bifurcation with a fine iris scissor (FST Inc, Foster City, CA) after anesthesia with a mixture of 10% ketamine hydrochloride (Sanofi-Ceva, Düsseldorf, Germany; 0.1 ml/100 g body weight) and Rompun (Bayer, Leverkusen, Germany; 0.05 ml/100 g body weight). For morphological analysis of degenerated nerves, the distal stumps of 1 mm length from lesion sites were discard and next 5 mm length distal stumps were collected at the indicated times.

### Human serum sampling and ELISA

The research protocol was approved by the institutional review board of Dong-A University (No. HR-004-02), Dong-A University Hospital (No. 13-042) and Samsung Medical Center (No. 2017-11-152) in Korea. Serum samples were collected from 36 CIDP (10 females, 26 males), 14 AIDP (3 females, 11 males), 20 AMAN (7 females, 13 males) and 39 CMT1a (17 females, 21 males) patients, as well as 20 healthy controls (14 females, 6 males) with informed consents of patients for the participation of the study. Detailed patient information was in Supplementary Table 1. Bloods were centrifuged at 3000 rpm for 10 min to separate the serum (plain tube, no anticoagulant), and the collected serum was stored at −80 °C until use. The diagnosis of CIDP and GBS (AIDP, AMAN) was made by the respective clinical and laboratory diagnostic criteria^30–32^. AMAN was further classified according to positive anti-ganglioside GM1 antibodies with enzyme-linked immunosorbent assay (ELISA) as we described previously for more accurate classification^33^. All serum samples of CIDP and GBS was collected by the Dong-A University Neuroimmunology Team (DAUNIT) for anti-ganglioside antibody testing with a presumptive diagnosis of immune-mediated neuropathy in collaboration with the Korean Inflammatory Neuropathy Consortium (KINC). KINC has systematically collected clinical and laboratory information of inflammatory neuropathy from university-based teaching hospitals in Korea. Serum of CMT1a patients were obtained from Samsung Medical Center.

Measurement of serum CXCL13 was performed with commercially available ELISA kits (#DY2408, R&D Systems). All tests were three experiments performed in triplicates according to the manufacturer’s instructions.

### Western blot analysis

For Western immunoblotting, cultured SCs were homogenized in the modified RIPA buffer. Lysates were fractionated on a SDS-PAGE gel and transferred to a nitrocellulose membrane (Millipore). The blotted membranes were blocked with 5% nonfat milk in Tris-buffered saline containing 0.05% Tween-20 (TBST) at room temperature for 1 hr, and the membranes were incubated overnight at 4 °C with primary antibodies diluted in TBST containing 3% nonfat milk. After 3 washes in TBST, the blots were reacted with HRP-conjugated secondary antibodies for 1 hr at room temperature and then washed again with TBST. For detection, an enhanced chemiluminescence-Western blot system (Amersham, Piscataway, USA) was employed and images were analyzed with LuminoGraph III (ATTO, Tokyo, Japan). The relative gray value of each target protein (gray value of a target band/gray value of β-actin band) was calculated with software CS analyzer (ATTO, Tokyo, Japan). For the quantitative analysis, at least three independent experiments were performed.

### Histological staining

Mouse sciatic nerves fixed in 4% paraformaldehyde were cryoprotected in a 20% sucrose solution. Cross sections with 14 μm thickness were made using a cryostat (Frigocut, Leica, Bensheim, Germany), and the section were stored in a deep freezer until use. The slides were blocked with PBS containing 0.2% Triton X-100 and 2% bovine serum albumin for 1 hr. The sections were then incubated with primary antibodies for 16 hr at 4 °C and washed three times with PBS. Next, the slides were incubated with Cy3- or Alexa 488-conjugated secondary antibody for 3 hr at room temperature, followed by DAPI staining for 30 min.

Four formalin-fixed, paraffin-embedded human sural nerves were provided by Severance Hospital of the Yonsei University Health System (Seoul), and consecutive 4 μm sections were made using a microtome. The research protocol was approved by the institutional review board of Severance Hospital (No. 4-2018-0317). The sections were then deparaffinized and rehydrated through graded ethanols. After antigen retrieval, the sections were blocked with 5% fetal bovine serum for 1 h at room temperature, and the same immunostaining protocols were applied as above.

For light microscopic analysis, stained sections were examined with a Zeiss AxioImager 2 microscope equipped with an ApoTome (Carl Zeiss, Göttingen, Germany). To count the number of DAPI-labeled nuclei or immunoreactive cells within the sciatic nerve sections, 8~10 images (500 μm × 650 μm) from three animal in each group were captured using Zen 2.3 proimaging software under a 403/1.2NA water immersion lens.

### Mouse cytokine array

Each sciatic nerve of NOD, B7-2KO, uncut C57BL/6 and injured C57BL/6 mice was homogenized in the modified RIPA buffer and the lysates were used for a cytokine antibody array designed to monitor the expression of 40 cytokines (Mouse Cytokine Array Panel A, #ARY006, R&D Systems). Briefly, lysates (300 μg) were incubated with an antibody-coated membrane on a rocking platform overnight. After three times wash with PBS to remove unbound materials, bound cytokines were detected via a sandwich ELISA format sequentially using the arrayed capture antibody and a cocktail of biotinylated detection antibodies. After three times wash with PBS, chemiluminescent detection reagents (Amersham) were added and then developed according to the manufacturer’s instructions.

### Statistical methods

Results were expressed as mean ± standard error of the mean (SEM). ELISA Data were analyzed using one-way analysis of variance followed by Kruskal-Wallis test and Sidak’s multiple comparisons test using GraphPad Prism version 6.01 (GraphPad Software Inc., La Jolla, CA). For statistical analysis of Western blot and of the number of immunoreactive cells in sections, unpaired Student’s *t*-test was performed. A value of *p* < 0.05 was considered significant.

## Supplementary information

Dataset 1
