# Supplementary material for: Serum CXCL13 reflects local B-cell mediated inflammatory demyelinating peripheral neuropathy
Source: Sci Rep. 2019 Nov 11;9:16535. doi: 10.1038/s41598-019-52643-2 (PMC6848485; doi:10.1038/s41598-019-52643-2)
Supplement: Supplementary file 1 — Dataset 1 [file 41598_2019_52643_MOESM1_ESM.pdf]

Title:
Serum CXCL13 reflects local B-cell mediated inflammatory demyelinating peripheral neuropathy

Running Head: CXCL13 in peripheral demyelinating neuropathy

Authors
Young Hee Kim, PhD,[1]* So Young Jang, PhD,[1] Yoon Kyung Shin, PhD,[1] Young Rae Jo, MD,[1] Byeol-A Yoon, MD,[1, 2,3] Soo Hyun Nam, PhD,[4] Byung-Ok Choi, MD, PhD,[4,5] Ha Young Shin, MD,[6] Seung Woo Kim, MD,[6] Se Hoon Kim, MD, PhD,[7] Jong Kuk Kim, MD, PhD,[1, 3#] and Hwan Tae Park, MD, PhD[1, 2#]

#Corresponding author
Hwan Tae Park MD, PhD,
Department of Molecular Neuroscience, Peripheral Neuropathy Research Center, Dong-A University College of Medicine,
Busan 49201, Republic of Korea
Fax. +82-51-247-3318, E-mail: phwantae@dau.ac.kr

Jong Kuk Kim MD, PhD
Department of Neurology, Peripheral Neuropathy Research Center, Dong-A University College of Medicine,
Busan 49201, Republic of Korea
Fax. +82-51-244-8668, Email: advania9@chol.com

Address
[1] Peripheral Neuropathy Research Center (PNRC), Dong-A University College of Medicine, Busan 49201, Republic of Korea

[2] Department of Molecular Neuroscience, Dong-A University College of Medicine, Busan 49201, Republic of Korea.

[3] Department of Neurology, Dong-A University College of Medicine, Busan 49201, Republic of Korea

[4] Department of Neurology, Sungkyunkwan University School of Medicine, Seoul 06351, Republic of Korea

[5] Stem Cell & Regenerative Medicine Institute, Samsung Medical Center, 81 Irwon-ro, Gangnam-gu, Seoul 06351, Republic of Korea

[6] Department of Neurology, Yonsei University College of Medicine, 50-1 Yonsei-ro, Seodaemun-gu, Seoul 03772, Republic of Korea.

[7] Department of Pathology, Yonsei University College of Medicine, 50-1 Yonsei-ro, Seodaemun-gu, Seoul 03772, Republic of Korea.

Supplementary Figure 1. Representative western blot showing CXCL13 expression. A cropped image was showin in Fig. 2C.

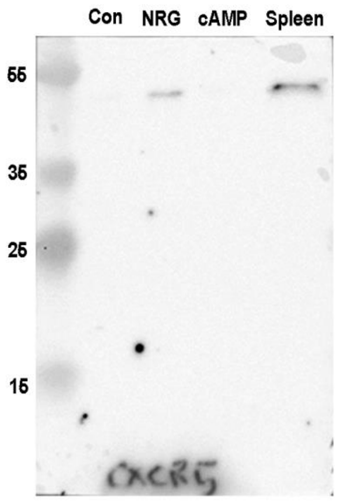

Supplementary Table 1. In the cases of AIDP and AMAN, serum was obtained within 2 weeks of symptom onset. In the case of CIDP, the disease duration is not known accurately, but all specimens were obtained before treatment. For CMT1A, the mean disease duration was 18.4 years and the disease duration ranged from 1 to 48 years.

	Sex		Age (Mean ± SD
	Female	Male	
**Control**	14	6	56.20 ± 12.28
**CIDP**	6	20	52.80 ± 18.08
**AIDP**	3	11	56.90 ± 13.89
**AMAN**	7	13	53.96 ± 12.44
**CMT1a**	17	21	36.23 ± 18.07
